# Platelet function studies in myeloproliferative neoplasms patients with *Calreticulin* or *JAK2*^V617F^ mutation

**DOI:** 10.1016/j.rpth.2023.100060

**Published:** 2023-01-31

**Authors:** Alexandre Guy, Khalil Helzy, Olivier Mansier, Jean-Claude Bordet, Etienne Rivière, Mathieu Fiore, Chloe James

**Affiliations:** 1 Laboratory of Hematology, University Hospital, Bordeaux, France; 2 University of Bordeaux, INSERM UMR 1034, “Biology of Cardiovascular Diseases”, Pessac, France; 3 Laboratory of Hematology, University Hospital of Lyon, Bron, France; 4 Internal Medicine and Infectious Diseases Unit, University Hospital, Bordeaux, France

**Keywords:** blood platelets, calreticulin, *JAK2*^V617F^ mutation, myeloproliferative neoplasms, platelet aggregation, thrombosis

## Abstract

**Background:**

*JAK2*^V617F^ and *Calreticulin (CALR)* mutations are the most frequent molecular causes of *Phi*-negative myeloproliferative neoplasms (MPN). Patients with *CALR* mutations are at lower risk of thrombosis than patients with *JAK2*^V617F^. We hypothesized that *CALR*-mutated blood platelets would have platelet function defects that might explain why these patients are at lower risk of thrombosis.

**Objectives:**

Our main objective was to explore and compare platelet function depending on the MPN molecular marker.

**Methods:**

We analyzed platelet function in 16 patients with MPN with *CALR* mutations and 17 patients with *JAK2*^V617F^ mutation and compared them with healthy controls. None of these patients was taking antiplatelet therapy. We performed an extensive analysis of platelet function and measured plasmatic soluble P-selectin and CD40L levels.

**Results:**

We observed significant defects in platelet aggregation, surface glycoprotein expression, fibrinogen binding, and granule content in platelets from patients with MPN compared with that in controls. Moreover, soluble CD40L and P-selectin levels were elevated in patients with MPN compared with that in controls, suggesting an *in vivo* platelet preactivation. Comparison of platelet function between patients with *CALR* and *JAK2*^V617F^ MPN revealed only minor differences in platelets from patients with *CALR*. However, these results need to be interpreted within the context of absence of an inflammatory environment that could impact platelet function during MPN.

**Conclusions:**

These results do not support the hypothesis that calreticulin-mutated platelets have platelet function defects that could explain the lower thrombotic risk of patients with *CALR*.

## Essentials

• Patients with MPN with *CALR* mutations are at lower risk of thrombosis than patients with *JAK2*^*V617F*^*.*
• Platelet function analyses were conducted in 16 patients with CALR MPN.
• Analysis revealed platelet function defects in patients with MPN compared with that in controls.
• We observed minor differences in platelets between patients with *CALR* and patients with *JAK2*^*V617F*^*.*

## 1 Introduction

*Phi*-negative myeloproliferative neoplasms (MPN) are malignant hemopathies characterized by clonal proliferation of hematopoietic cells, owing to the acquisition of driver mutations in hematopoietic stem cells, and include polycythemia vera (PV), essential thrombocythemia (ET), and primary myelofibrosis. Three main mutations, ie, *JAK2*^V617F^, *MPL*, and *CALR*, are found in more than 90% of patients with MPN and induce a constitutive activation of the JAK-STAT pathway. In 2013, the discovery of mutations affecting calreticulin [1,2], a protein so far mainly known for its function within the endoplasmic reticulum (ER), raised new questions in our understanding of MPN. Calreticulin is a 46-kDa protein composed of 3 domains. The N- and P-domains contain the lectin and ERp-57 binding sites, allowing calreticulin to play its role of chaperone [3,4] because it contributes to better folding of neosynthesized glycoproteins. Calreticulin is also a major protein in calcium homeostasis—thanks to the C-domain that contains many calcium-binding sites, allowing its storage/buffering within the ER. More than 50 *CALR* mutations have been previously described, with 2 of them, ie, *CALRdel52* and *CALRins5*, representing more than 80% of cases [1,2]. All *CALR* mutations affect the C-domain of the protein resulting in the same 36 amino acid terminal sequence and the loss of several calcium-binding sites. Interestingly, it has been shown that CALR mutants can activate the JAK/STAT pathway by binding to the N-glycosylated residue of the thrombopoietin receptor MPL [[5], [6], [7], [8]].

Disease evolution of MPN is marked by the onset of several complications, among which thrombosis is the most frequent. It occurs in approximately 30% of PV [9] and 20% of ET [10] at diagnosis and in more than 10% during the follow-up of the disease [9,10]. The 2 main risk factors for thrombosis are age older than 60 years and a history of thrombosis. Surprisingly, although *CALR* and *JAK2*^V617F^ mutations act on the same intracellular signaling pathways, the thrombotic risk is significantly lower in patients carrying *CALR* mutations compared with *JAK2*^*V617F*^ in essential thrombocythemia (ET) [1,11]. The reason for such a difference is yet to be investigated.

Many studies have sought to explore the roles of the different blood cells in the pathogenesis of thrombosis during MPN [12]. Because platelets play a critical role in thrombus formation, they require particular attention when exploring the mechanisms of thrombogenicity in MPN. Knowing the key role of calcium in platelet activation, we hypothesized that the potential dysregulation of calcium homeostasis induced by *CALR* mutations could disturb platelet function, explaining the relative decreased risk of thrombotic complications in these patients. In this study, our objective was to explore and compare platelet functions in patients with MPN carrying *CALR* or *JAK2* mutations without taking any antiplatelet therapy.

## 2 Methods

### 2.1 Study population

Sixteen patients with *CALR* and 17 patients with *JAK2*^V617F^ MPN without ongoing antiaggregatory drugs were prospectively enrolled in the University Hospital of Bordeaux between 2018 and 2020 after having obtained their informed consent. In total, 11 patients were treated using hydroxyurea. All investigations were approved by the local ethics committee. Diagnoses of MPN were made according to WHO 2008 criteria. Because we were concerned that a sufficient proportion of platelets would be mutated for *CALR* or *JAK2*^V617F^, we chose to only include patients with an allele burden greater than 10%. The results were compared with those obtained from 10 different healthy volunteers.

### 2.2 Collection of blood samples

For platelet count, blood was collected on sodium ethylene diamine tetraacetate-anticoagulated tubes. Platelet counts were obtained using the Beckman DXH 800 hematology analyzer (Beckman Coulter), which is used routinely. For platelet function assays and electron microscopy, blood was collected on citrate tubes containing 0.109 molar sodium citrate and tubes containing acid citrate dextrose. To avoid platelet activation, samples were analyzed within 2 hours after sampling. Dry tubes for serum collection were also used.

### 2.3 Light transmission aggregometry

Platelet aggregation was tested in citrated platelet-rich plasma (PRP) using 2.5 μM adenosine diphosphate (ADP; Calbiochem), 1 mM arachidonic acid (Nu Chek Prep), 1 μg/mL Horm equine tendon collagen (Nycomed, Pharma), 10 μM Thrombin Receptor Activating Peptide-14 (TRAP14-mer; Neosystem SA), 4 μM epinephrine (Sigma-Aldrich), and 5 μM ionophore 23187 (Calbiochem) in an APACT-4004 aggregometer (Elitech) according to standard procedures [13]. Native PRP concentration was adjusted if the platelet count was higher than 600G/L to reach a platelet count of 500 G/L.

### 2.4 Platelet adenosine triphosphate release study

Platelet adenosine triphosphate (ATP) secretion was described previously [14]. Briefly, ATP secretion was recorded in real time at 37 °C with stirring on a dual-channel Chrono-Log aggregometer (Chronolog Corp) using 5 μM ADP (Chronolog Corp), 1 μg/mL collagen (Chronolog Corp), and 10 μM TRAP-6 (Hart Biologicals Ltd). Platelet secretion was determined by measuring the release of ATP using luciferin/luciferase reagent (Kordia). The results were expressed as nmol of secreted ATP.

### 2.5 Platelet flow cytometry studies

Surface expressions of the main platelet glycoproteins (GPs) GPIb, α_IIb_β_3_, and P-selectin were measured in PRP (250 × 10^9^/L) using a platelet calibrator kit (Biocytex) with specific monoclonal antibodies, according to the manufacturer’s instructions. Briefly, after sample stabilization at room temperature for 1 hour, platelets were stained for 20 minutes before cytometry analysis. Readings were taken before and after activation with 60 μM TRAP. Results were expressed as number of sites derived from mean fluorescence intensities.

Platelet fibrinogen binding was measured using PRP adjusted at 250 × 10^9^/L and incubated with Alexa Fluor 488–labeled human fibrinogen (Molecular probes) after activation by 10-μM ADP.

Before stimulation, platelets were washed using 2 centrifugations (10 minutes, 2600 rpm) and by the addition of washing buffer (Tyrode buffer, glucose, calcium chloride, and apyrase). Washed platelets were stimulated, or not, with 5-μM ionophore-A23187 free acid (5 μM) (Calbiochem) for 5 minutes. The samples were then incubated with FITC-conjugated annexin V (BD Pharmingen Inc) for measuring phosphatidylserine (PS) expression. A Cytomics FC500 flow cytometer (Beckman Coulter) was used for all experiments.

### 2.6 Whole mount electron microscopy

Small drops of PRP were deposited on formvar-coated grids (Electron Microscopy Sciences) for 1 to 5 minutes, rinsed in a drop of distilled water (10-15 seconds), dried from the edge with pieces of a filter paper and air dried during 1 minute with gentle shaking and without further chemical fixation or poststaining with contrasting agents. Platelet dense granules were counted with a JEOL JEM1400 transmission electron microscope equipped with a Gatan Orius 600 camera and DigitalMicrograph software [15].

### 2.7 Soluble markers of platelet activation

Soluble P-selectin was measured in patients’ sera using the Quantikine Human P-selectin ELISA kit (R&D systems), and soluble CD40L with the Invitrogen Human CD40L ELISA Kit (ThermoFischer Scientific), according to the manufacturer’s instructions.

### 2.8 Statistical analysis

The presence or absence of normality was tested for all the parameters analyzed. Comparisons between the 3 groups were made using 1-way analysis of variance (followed by analysis of multiple comparisons with Tukey’s test) or the Kruskal–Wallis test (followed by analysis of multiple comparisons with Dunn’s test). Owing to the absence of normality distribution for the groups analyzed, comparisons between 2 groups required Mann–Whitney U-tests. All results, comparisons, and graphs were performed with the statistical software GraphPad Prism 9. A *P* value < .05 was considered statistically significant.

## 3 Results

### 3.1 Characteristics of patients with MPN

Thirty-three patients with *CALR* or *JAK2*^V617F^ mutations were included. None of the patients included were receiving antiplatelet agents. Overall, 16 of 33 (48.5%) patients were included during the first year of MPN diagnosis. Sixteen patients carried a *CALR* mutation and were diagnosed with ET. Seventeen patients were *JAK2*^V617F^ positive, including 9 ET, 6 PV, and 2 prefibrotic myelofibrosis. Bone marrow examination was performed in 10 patients: 2 had a diagnosis of preprimary myelofibrosis and the 8 others were diagnosed as ET with no fibrosis. The ages of patients with *CALR*+ (56.8 years old ± 21.8) and *JAK2*^V617F^ + MPN (65.6 years old ± 11) were not different. Male-to-female ratio was similar between the 2 groups. All patients and controls were Caucasians. Allele burden was similar between patients with *CALR*+ (mean: 31.2%, min: 11%, max: 48%) and *JAK2*^V617F^+ (mean: 45%, min: 15%, max: 88%). Twelve patients (37.5%; 6 patients with *CALR*+ and 6 patients with *JAK2*^V617F^ + MPN) were treated with cytoreduction. Patients with *CALR+* MPN had an increased platelet count (793 × 10^9^/L ± 104) compared with patients with *JAK2*^V617F^+ MPN (PLT = 555 × 10^9^/L ± 75.8) (Table 1). The results were compared with those of the controls with the analysis of 10 healthy volunteers. Seven men and 3 women were studied with a mean age of 34 years (SD: 6.9) and a mean platelet count of 248 G/L (SD: 24).

**Table 1:** Main characteristic of patients with MPN and controls.

Main clinical and biological characteristics	Control (*n* = 10)	*CALR*+ patients (*n* = 16)	*JAK2*V617F+ patients (*n* = 17)	*P*
Age (y) (mean ± SD)	34 ± 6.9	56.8 ± 21.8	65.6 ± 11	**<.001**
Sex (*n* =)	M = 7 F = 3	M = 5 F = 11	M = 10 F = 7	.07
Ethnicity	Caucasian: *n* = 10	Caucasian: *n* = 16	Caucasian: *n* = 17	
MPN type (*n* =)	NA	ET = 16	ET = 9 PV = 6 Pre-PMF = 2	
Mutation type (*n* =)	NA	Type 1 = 7 Type 2 = 7 Other = 2	NA	
Allele burden (%, min-max)	NA	31.2 (11-48)	42 (15-88)	.23
Cytoreductive therapy (*n* =)	NA	None = 10 Hydroxyurea = 5 Anagrelid = 1	None = 11 Hydroxyurea = 6	
Platelet count (G/L)	248 ± 24	793 ± 104	555 ± 75.8	**.002**
(mean ± SD)				
Leukocyte count (G/L)	NA	7.0 ± 2.6	8.1 ± 2.3	.25
(mean ± SD)				
Neutrophils count (G/L)	NA	4.8 ± 2.4	5.7 ± 0.5	.34
(mean ± SD)				
Hemoglobin (g/dl)	NA	13.1 ± 1.6	14 ± 1.6	.13
(mean ± SD)				

Statistical significance assessed by using the Student’s *t*-test for allele burden and platelet count and by using the Fisher exact test for gender repartition. Statistically significant *P* values are indicated in bold.

*CALR*, calreticulin; ET, essential thrombocythemia; F, female; M, male; MPN, myeloproliferative neoplasms; NA, not applicable; PV, polycythemia vera; pre-PMF, pre-fibrotic primary myelofibrosis.

### 3.2 Patients with MPN have moderate platelet aggregation defects

**Figure 1:**
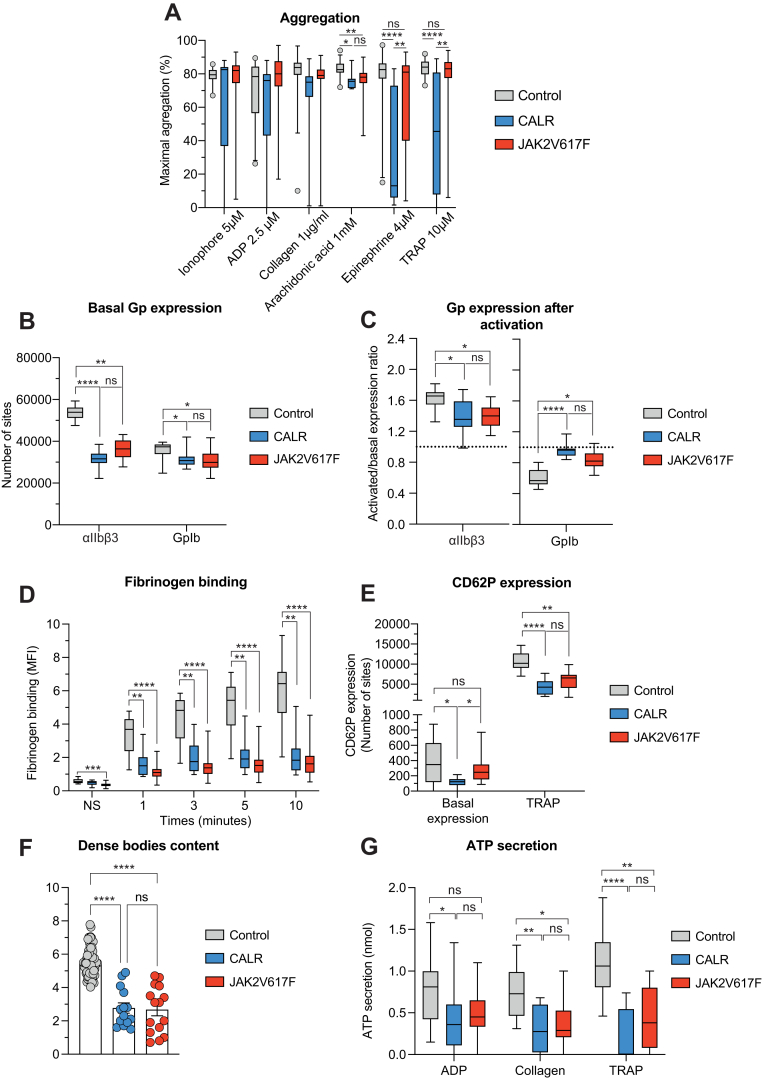
*In vitro* assessment of platelet function in platelets from *CALR-* and *JAK2*^V617F^-mutated patients. (A) Light transmission aggregometry in platelets from 33 patients with *CALR-* and *JAK2*^V617F^ mutation. ADP: adenosine diphosphate; TRAP: thrombin receptor activating peptide. Data are presented as boxes with 5% and 95% percentiles. Boxes show the first and third quartiles. Whiskers indicate 5% and 95% percentiles. Statistical significance assessed by using 1-way analysis of variance followed by posthoc Dunn’s test. ∗*P* < .05; ∗∗*P* < .01; ∗∗∗*P* < .001; ∗∗∗∗*P* < .0001. (B) α_IIb_β_3_ and GPIbα expression at resting state; (C) Ratios of α_IIb_β_3_ and GPIbα expressed after platelet activation with TRAP 60 μM. Data are presented as boxes with 5% and 95% percentiles. Statistical significance was assessed using the 1-way analysis of variance followed by posthoc Dunn’s test. ∗*P* < .05; ∗∗*P* < .01; ∗∗∗*P* < .001; ∗∗∗∗*P* < .0001. Analysis realized in platelets from 31 patients. (D) Fibrinogen binding in platelets from patients with *CALR-* and *JAK2*^V617F^ mutation. Fibrinogen binding was evaluated using flow cytometry at baseline and at different time points after ADP activation. Data are presented as boxes with 5% and 95% percentiles. Statistical significance was assessed using 1-way analysis of variance followed by posthoc Dunn’s test. ∗∗*P* < .01; ∗∗∗*P* < .001; ∗∗∗∗*P* < .0001. Analysis realized in platelets from 33 patients. (E) CD62P expression in platelets from *CALR-* and *JAK2*^V617F^-mutated patients at resting state and after platelet activation with TRAP 60 μM. Data are presented as boxes with 5% and 95% percentiles. Statistical significance was assessed using the 1-way analysis of variance followed by posthoc Dunn’s test. ∗*P* < .05; ∗∗*P* < .01; ∗∗∗∗*P* < .0001. Analysis realized in platelets from 31 patients. (F) Evaluation of δ-granules content in platelets from patients with *CALR* and *JAK2*^V617F^ mutation by analysis of dense body content by using electronic microscopy. Data are presented as mean and SEM. Analysis realized in platelets from 30 patients. (G) Evaluation of δ-granules content in platelets from patients with *CALR* and *JAK2*^V617F^ mutation ATP secretion measurement in the presence of different agonists: ADP 5 μM, collagen 1 μg/mL, and TRAP 10 μM. Data are presented as boxes with 5% and 95% percentiles. Statistical significance was assessed using 1-way analysis of variance followed by posthoc Dunn’s test. ∗*P* < .05; ∗∗*P* < .01; ∗∗∗∗*P* < .0001. Analysis realized in platelets from 33 patients.

We first assessed platelet function using light transmission aggregometry with 6 different agonists. We did not observe any difference in response to 5-μM ionophore, 2.5-μM ADP, and 1 μg/mL collagen, whereas in response to arachidonic acid, platelet aggregation was significantly lower in patients with MPN compared with controls (Figure 1A). Interestingly, epinephrine- and TRAP-induced platelet aggregation was significantly lower in patients with *CALR+* compared with control and patients with *JAK2*^V617F^+ (Figure 1A). This result was confirmed when we separately analyzed only patients with ET (9 *JAK2*^*V617F*^ and 16 *CALR*) (Supplementary Fig. S1).

### 3.3 Platelet surface glycoprotein expression is impaired in patients with MPN

We then wondered whether there were differences in surface glycoprotein expression. At baseline, α_IIb_β_3_ and GPIb expressions were significantly reduced in blood platelets from patients with *CALR*+ and *JAK2*^V617F^+, without difference between blood platelets from patients with *CALR*+ and *JAK2*^V617F^+ MPN (Figure 1B). After activation, we observed a similar and significant defect in α_IIb_β_3_ expression and GPIb internalization in both MPN groups compared with controls (Figure 1C). A subanalysis of patients with ET (9 *JAK2*^*V617F*^ and 16 *CALR*) showed slightly different results (Supplementary Fig. S1) with a significant decrease in GpIIbIIIa expression on resting platelets and a significant decrease in Gp1b internalization after activation. Altogether, these results showed similar expression defects of the main surface glycoproteins between patients with *CALR* and *JAK2*^V617F^+.

### 3.4 Fibrinogen-binding defects in MPN platelets

Fibrinogen binding is a marker of α_IIb_β_3_ activation with a switch to a high-affinity conformation, allowing blood platelets to aggregate. We explored fibrinogen binding to α_IIb_β_3_ at different time points after stimulation with 10-μM ADP. Fibrinogen binding was similarly and significantly impaired in both MPN groups compared with controls, at all time points analyzed (Figure 1D), which may be related to decreased expression of α_IIb_β_3_ expression (Figure 1B).

### 3.5 Platelet granule defects

To study α-granules, we measured P-selectin (CD62P) on resting and activated platelets. Platelets from patients with CALR-positive mutation showed a significant reduction in baseline P-selectin expression compared with platelets from controls and patients with *JAK2*^V617F^-positive MPN. The difference between *CALR* and *JAK2*^V617F^ patients’ platelets was not significant when we restricted our analysis to patients with ET (Supplementary Fig. S1). After platelet activation, P-selectin expression was significantly reduced in both groups of patients with MPN without difference between platelets from patients with *CALR*-positive and *JAK2*^V617F^-positive MPN (Figure 1E).

We then studied platelet dense granule content using electron microscopy, showing a reduction of dense body number in patients with MPN, without differences between *CALR* and *JAK2*^V617F^ groups (Figure 1F). Because ATP is a large component of dense bodies, the measure of its release was used to analyze dense granule content. We observed a significant defect in ATP secretion in patients with *CALR+* MPN when platelets were stimulated by TRAP, ADP, and collagen. Similar results were observed in platelets from patients with *JAK2*^V617F^ and a decreased ATP secretion in the presence of collagen and TRAP. No difference was observed between the groups *JAK2*^V617F^ and *CALR* (Figure 1G). Altogether, these results suggest a platelet defect in α- and δ-granules in patients with MPN, without any difference between platelets from patients with *CALR*- and *JAK2*^V617F^-positive MPN.

### 3.6 Platelets from patients with *JAK2*^V617F^*+* mutation have increased procoagulant activity compared with controls

**Figure 2:**
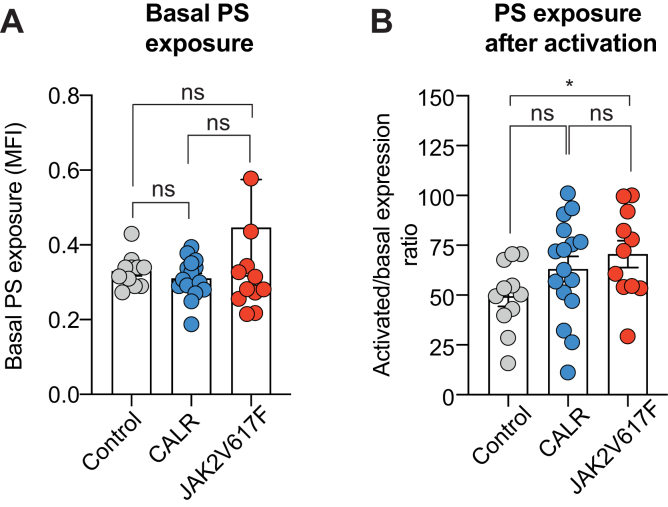
Phosphatidylserine exposure in platelets from patients with *CALR* and *JAK2*^V617F^ mutation. (A) PS exposure at resting state; (B) PS exposure after platelet activation with ionophore 5 μM. The PS induction ratio is calculated as the ratio of PS exposure after stimulation to baseline. Data are presented as mean and SEM. Statistical significance was assessed by 1-way analysis of variance followed by posthoc Dunn’s test. ∗*P* < .05. Analysis realized in platelets from 29 patients.

During platelet activation, the intracellular release of calcium activates the enzymes responsible for phospholipid externalization. Thereby, phosphatidylserine is translocated to the outer membrane surface, allowing binding of coagulation factors. We thus measured phosphatidylserine exposure by using annexin V, before and after platelet activation with 5 μM ionophore. At baseline, there was no difference in phosphatidylserine exposure between the 3 groups (Figure 2A), whereas after platelet activation, phosphatidylserine exposure was significantly higher in the *JAK2*^V617F^ group in comparison with controls (Figure 2B), suggesting an increased procoagulant activity in this group. We did not observe differences in PS exposure between platelets from patients with *CALR*+ and *JAK2*^V617F^+.

To note, we did not observe any significant differences between patients with low- and high-allele burdens, either in the *JAK2*^V617F^ or *CALR* group. We also found no difference in platelet aggregation, glycoprotein expression, fibrinogen binding, dense body content, and ATP secretion (Supplementary Fig. S2).

### 3.7 Patients with MPN have increased soluble markers of platelet activation

**Figure 3:**
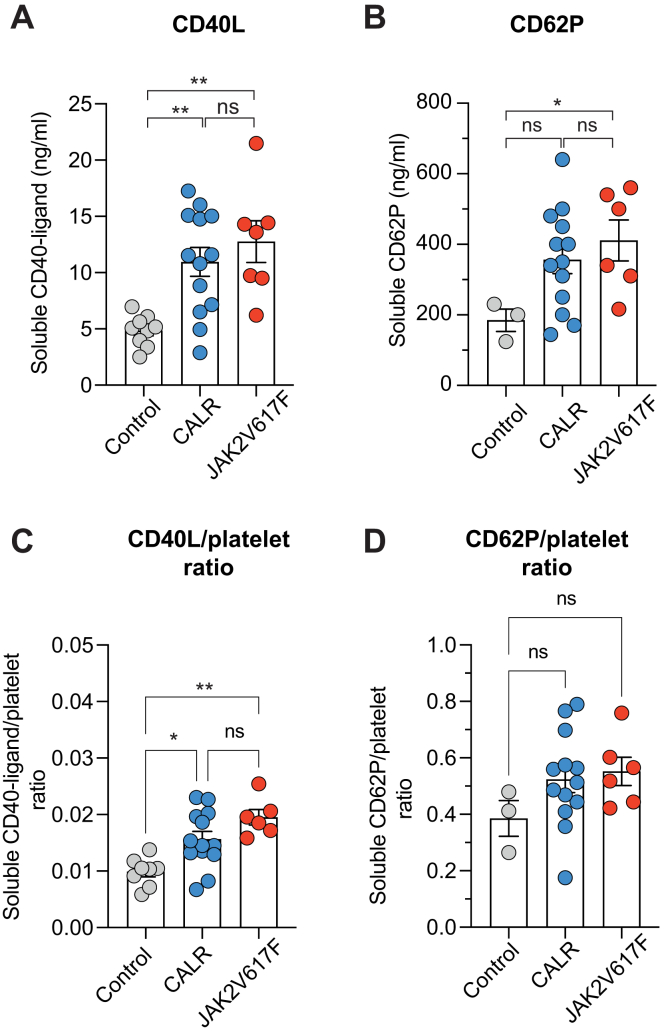
CD40L and sCD62P concentrations in sera from patients with *CALR* and *JAK2*^V617F^. (A) Soluble CD40-ligand concentration. Statistical significance was assessed using 1-way analysis of variance followed by posthoc Dunn’s test. ∗∗*P* < .01. Data are presented as mean and SEM. Analysis realized with 20 patients. (B) Soluble CD62-P concentration. Statistical significance was assessed using Kruskal–Wallis test followed by posthoc Dunn’s test. Data are presented as mean and SEM. Analysis realized with 19 patients. (C) CD40-L/platelet ratio analysis. Statistical significance was assessed using Kruskal–Wallis test followed by posthoc Dunn’s test. Data are presented as mean and SEM. Analysis realized with 20 patients. (D) CD62-P/platelet ratio analysis. Statistical significance was assessed by using Kruskal–Wallis test followed by posthoc Dunn’s test. Data are presented as mean and SEM. Analysis realized with 19 patients.

To evaluate *in vivo* platelet activation, P-selectin and CD40L amounts were measured in patient sera. This method offers a reliable evaluation of the platelet activation state *in vivo*, whereas the upper functional tests explore *ex vivo* platelet reactivity. Soluble CD40L concentration (Figure 3A) was significantly higher in *CALR* and *JAK2*^V617F^ groups, suggesting a previous platelet activation state in our patients. We also observed increased soluble P-selectin (Figure 3B) concentration in patients with *JAK2*^V617F^+. In patients with *CALR*+, soluble P-selectin concentration was also increased but without statistical significance (*P* = .06). To analyze the platelet activation status and because platelet count differs between controls and patients with MPN, we then analyzed the ratio between CD40L or P-selectin and the platelet count. The soluble CD40L/platelet ratio was increased in patients with *CALR* and *JAK2*^V617F^, confirming the *in vivo* platelets activation (Figure 3C). We did not observe a statistically significant increase of soluble P-selectin/platelet ratio, possibly owing to the low number of controls analyzed for this marker (Figure 3D). Finally, we did not observe any difference between *CALR* and *JAK2*^V617F^ groups regarding CD40L or soluble P-selectin concentration.

### 3.8 Hydroxyurea does not affect most platelet function tests but reduces procoagulant activity in patients with MPN

**Figure 4:**
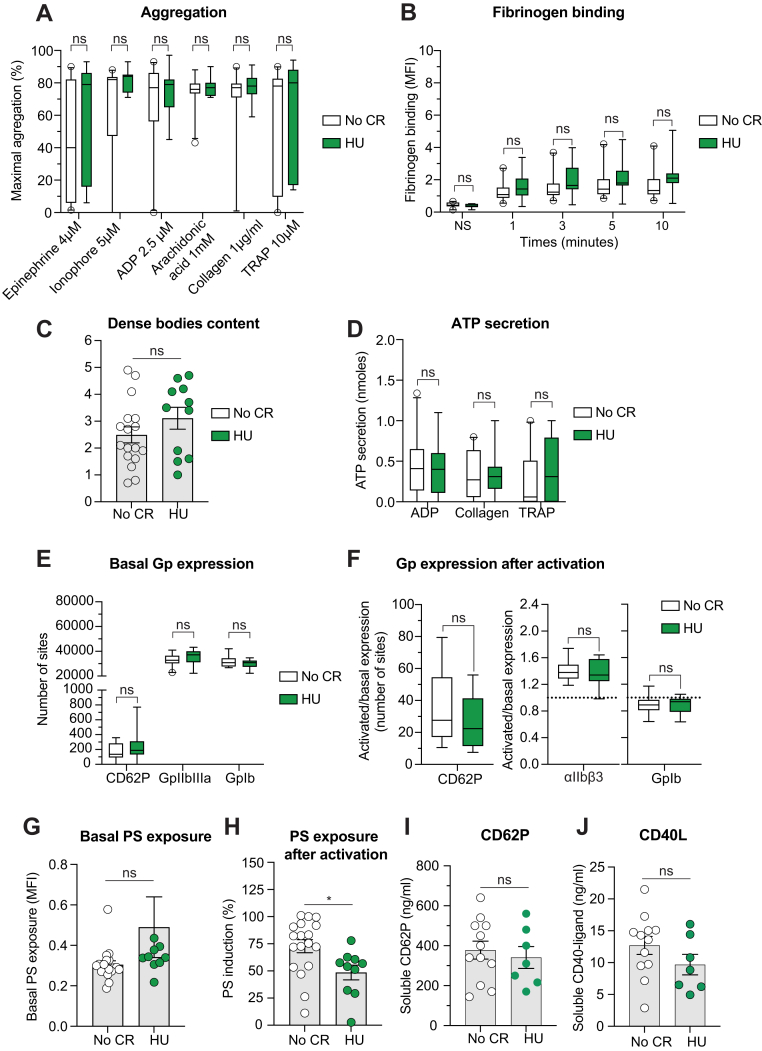
Comparison of platelet function in patients treated by hydroxyurea and patients without cytoreductive therapy. (A) Platelet aggregation in presence of epinephrine 4 µM, ionophore 5 µM, ADP 2,5 µM, arachidonic acid 1mM, collagen 1 µg/ml, TRAP 10 µM; (B) Fibrinogen binding after ADP activation; (C) Dense bodies content analysis; (D) ATP secretion evaluation; (E) Glycoprotein expression at baseline and (F) after platelet activation; (G) Basal PS exposure; (H) PS exposure after platelet activation. The PS induction ratio is calculated as the ratio of PS after stimulation to baseline; (I) CD62P concentration and (J) CD40L concentration. Statistical significance assessed by Mann-Whitney test. ∗ = *P* < .05. Data are presented as boxes with 5% and 95% percentiles or mean and SEM.

Treatment with hydroxyurea (HU) is known to decrease the thrombotic risk in patients with MPN, mainly through decreased production of blood cells. To test whether treatment with HU could influence platelet function, we analyzed separately platelet function results in the 11 patients with MPN taking HU and in 21 patients without cytoreductive treatment whatever their molecular defects. These 2 groups were similar in *JAK2*^V617F^/*CALR* status, age, gender repartition, MPN type, or allele burden. As expected, patients with HU had a significantly lower platelet count (Table 2). In most platelet function assays, we did not observe any difference between treated and untreated patients **(**Figures 4A–G**)**. Only phosphatidylserine exposure was reduced in the group of patients with HU, in favor of a lower procoagulant activity with this treatment (Figure 4H). Finally, there was no difference in P-selectin or CD40L concentrations between patients treated with HU and untreated patients (Figures 4I–J).

**Table 2:** Main characteristics of hydroxyurea-treated and nontreated patients with MPN.

Main clinico-biological characteristics	No cytoreductive therapy (*n* = 20)	Hydroxyurea (*n* = 11)	*P*
Age (y) (mean ± SD)	60 ± 17.7	64 ± 17.5	.41
Sex (*n* =)	M = 10 F = 10	M = 5 F = 6	>.99
MPN type (*n* =)	ET = 16 PV = 2 PMF = 2	ET = 7 PV = 2 PMF = 1	
Mutation type (*n* =)	*CALR* = 10 *JAK2*^V617F^ = 10	*CALR* = 5 *JAK2*^V617F^ = 6	>.99
Allele burden (%, min-max)	34.58 (11-85)	39.4 (5-78)	.56
Platelet count (G/L) (mean ± SD)	797.2 ± 391.5	435.3 ± 65.4	**.009**
sCD62P concentration (ng/mL) (mean ± SD)	377.8 ± 45	340.9 ± 54	.66
CD40-ligand concentration (ng/mL) (mean ±SD)	12.7 ± 4.9	9.7 ± 1.6	.18

Statistical significance assessed by using Student’s *t*-tests or Mann–Whitney U-tests for age, allele burden, platelet count, and sCD62P and CD40-L concentrations. Statistical significance assessed by using Fisher exact tests for gender and mutation type repartition. Statistically significant *P* values are indicated in bold.

*CALR*, calreticulin; ET, essential thrombocythemia; F, female; M, male; MPN, myeloproliferative neoplasms; NA, not applicable; PV, polycythemia vera; PMF, fibrotic primary myelofibrosis.

## 4 Discussion

Patients carrying *CALR* mutations are known to be at lower risk of thrombosis than patients with *JAK2*^V617F^. This study focused on platelet functions to investigate whether specific defects might explain this difference. Because calreticulin is a major protein in calcium regulation, we initially hypothesized that *CALR* mutations could impair platelet reactivity and reduce thrombotic risk in patients with *CALR* compared with those with *JAK2*^V617F^. We also aimed to compare platelets from *CALR* and *JAK2*^V617F^+ patients with platelets from healthy controls.

We here observed that *in vitro* platelet function was impaired in patients with MPN, with arguments for *in vivo* platelet preactivation. The comparison of patients with *CALR* and *JAK2*^V617F^+ highlighted moderate differences in platelet function, not suggesting a crucial role for platelets in the difference in thrombotic risk.

Our results revealed that most of platelet function studies were defective in patients with MPN compared with healthy controls. These defects include storage pool deficiency, impaired aggregation in response to agonists, and decrease in glycoprotein expression and fibrinogen binding after platelet activation. We did not observe any statistically significant differences between patients with low- and high-allele burden, either in the *JAK2*^V617F^ or *CALR* group. Nevertheless, allele burden was quantified in leukocytes but not in platelets. As a recent study reported that the allele burden can be different between platelets and granulocytes [16], we cannot definitively conclude that the allele burden in platelets does not correlate with the platelets function defect we observed. Our observations of defective platelet function in patients with MPN may seem counterintuitive, given the prothrombotic state during MPN, where one would have expected hyperreactive platelets. These *in vitro* defects were also reported in a number of previous works. For instance, impairment of platelet aggregation [17] and the defects of membrane glycoprotein expression [18,19] and granule content [20,21] have been previously described. A study reported abnormalities in arachidonic acid metabolism [20], which is consistent with our results that showed decreased aggregation in MPN platelets after activation with arachidonic acid. Recently, a study also revealed platelet dysfunction in *JAK2*^V617F^ transgenic mice [22].

The reasons for the *in vitro* MPN platelet function defects observed in our study, although consistent with the literature, deserve to be investigated. One hypothesis is that these platelets could be exhausted platelets that have already been activated and therefore have already released their content. The defect we observed *in vitro* would thus be an after-effect of an *in vivo* platelet preactivation. In line with that, platelet activation products were shown to be increased during MPN, such as platelet factor 4, β-thromboglobulin [23], urinary thromboxane metabolites [24], and soluble P-selectin or CD40 [25]. Our study confirms these observations as we report higher concentrations of soluble CD40L and P-selectin within *CALR* and *JAK2*^V617F^ groups than in controls. This *in vivo* platelet activation may be due to intrinsic platelet activation because of JAK-STAT pathway activation in megakaryocytes. It is known that the JAK2-STAT3 pathway is involved in collagen-induced platelet activation [26], and it has been demonstrated that a constitutive Src kinase preactivation is involved in platelet hyperreactivity in patients with MPN [27]. It has also been shown, using a mouse model allowing the expression of the human JAK2V617F, that *JAK2*^V617F^ megakaryocytes (MKs) display increased migration and proplatelet formation. In the same study, the authors demonstrated significant changes in RNA expression in *JAK2*^V617F^ MKs, notably in the cytoskeleton assembly and apoptosis pathways [28]. This could also be a consequence of platelet activation by inflammatory molecules or increased thrombin generation. Finally, it suggests that *CALR* and *JAK2*^V617F^ platelets are equally activated *in vivo*.

Given the participation of platelets in coagulation pathway activation, we studied platelet procoagulant activity and observed that the procoagulant activity (explored by the measurement of phosphatidylserine exposure) was higher in the *JAK2*^V617F^ group only in comparison with controls. Besides, the importance of investigating procoagulant activity was demonstrated in a recent work conducted in patients with ET that compared phosphatidylserine exposure on blood cells from different mutational subtypes. Patients with *JAK2*^V617F^ had a higher phosphatidylserine exposure than *CALR* and triple-negative groups, which was correlated with more hypercoagulability and could possibly explain the higher thrombotic risk in the *JAK2*^V617F^ group [29]. These results suggest that *JAK2*^V617F^ platelets could trigger coagulation activation in a greater extent than CALR platelets.

Because the discoveries of driver mutations are relatively recent, most of the studies that focuses on platelet function in patients with MPN have been performed regardless of the mutation type. It is essential to distinguish the different mutational subtypes because clinical studies revealed differences in thrombotic risk depending on the driver mutation [9,10]. Our investigations here report a defective platelet aggregation in the presence of epinephrine and TRAP and slight differences in glycoprotein expression in platelets from patients with *CALR* compared with platelets from patients with *JAK2*^V617F^ mutation. Analysis of all MPN patients showed a significant decrease in basal CD62P expression in platelets from patients with *CALR* compared with that from patients with *JAK2*^V617F^ mutation, and subanalysis in patients with ET showed a significant decrease in basal GpIIbIIIa expression and a significant decrease in Gp1b internalization after activation. However, all the other tests showed comparable *in vitro* defects between platelets from patients with *CALR* and *JAK2*^V617F^ mutation. The study of platelet characteristics from patients with *CALR* and *JAK2*^V617F^-positive ET has been performed in a recent study. On the basis of activation and adhesion assays, they concluded to less reactivity of *CALR*-mutated platelets in comparison with *JAK2*^V617F^ platelets, with the defect of CD62P and α_IIb_β_3_ expressions after ADP stimulation [30]. However, this study has a major limitation because more than 90% of patients were taking antiplatelet therapy. Contrary to the study by Hauschner et al. [30], we did not observe decreased CD62P expression or α_IIb_β_3_ expression after stimulation, but we did not use the same agonist. Nevertheless, the differences we here report are minor, though significant, as the only tests that were different are maximal aggregation after stimulation with epinephrin and TRAP (but not with other inductors) and slight differences in glycoprotein expression. Altogether, we do not think that such slight differences could explain the difference in thrombotic risk observed in clinical studies between patients with *CALR* and *JAK2*^V617F^ mutation. To our knowledge, our study is first analyzing *CALR-* and *JAK2*^V617F^-mutated platelets of patients free from antiplatelet drugs. This inclusion criterion appears essential because these drugs affect platelet functions.

Regarding cytoreductive treatments, few studies have explored their potential effect on platelet function. One compared patients receiving HU with untreated ones and did not find any difference in P-selectin expression [31], whereas the other used the markers of *in vivo* clotting formation (levels of prothrombin fragment and thrombin–antithrombin complex) and described no differences as well [32]. A recent study analyzed platelet characteristics after aspirin uptake in HU or pegylated interferon alpha (peg-INF)-treated patients with ET. The authors observed that patients treated with peg-IFN had an increased aspirin-mediated platelet inhibition than patients treated with HU [33]. Our comparison of patients with MPN with and without HU showed a reduction in platelet procoagulant activity in the treated group after platelet stimulation by ionophore. This result is consistent with another study that described a decrease in thrombin generation when patients suffering PV and ET were treated by HU [34].

Our study has some limitations. The first is the presence of hydroxyurea-treated patients with MPN (11/28). Indeed, hydroxyurea has potential anti-inflammatory effects that could thus modify platelet functions. Second, we did not analyze the extrinsic factors that may modify platelet functions, such as inflammatory cytokines. Indeed, it is known that patients with MPN do have a proinflammatory environment that participates in the physiopathology of the disease [35].

In conclusion, our study showed global *in vitro* defect of platelet function in patients with *CALR* and *JAK2*^V617F^ compared with controls. Interestingly, we here provide an extensive analysis of platelets from patients with *CALR* mutation, highlighting the presence of *in vitro* defective function, in a same extent than patients with *JAK2*^V617F^-positive mutation. Finally, soluble markers of platelet activation were increased in both MPN groups, suggesting that the *ex vivo* platelet function defects observed could be due to previous *in vivo* activation, either at the megakaryocyte level or extrinsic factors such as inflammatory molecules or increased thrombin generation.

## Supplementary material

Supplementary Figure 1 Analysis of platelet function in ET patients. (A) Platelet aggregation in presence of epinephrine 4 μM, ionophore 5 μM, ADP 2,5 μM, arachidonic acid 1 mM, collagen 1 μg/ml, TRAP 10 μM; B) Glycoprotein expression at baseline and (C) after platelet activation; (D) Fibrinogen binding after ADP activation; (E) CD62P expression at basal level and after platelet activation; (F) Dense bodies content analysis; (G) ATP secretion evaluation; (H) Basal PS exposure (I) and after platelet activation. (J) CD40-L/platelet ratio analysis. Statistical significance assessed by Kruskal-Wallis test followed by post-hoc Dunn’s test. Data are presented as mean and SEM. (K) CD62-P/platelet ratio analysis. Statistical significance assessed by Kruskal-Wallis test followed by post-hoc Dunn’s test. Data are presented as mean and SEM.

Supplementary Figure 2 Platelet function testing in patients with low or high allelic burden. (A) Platelet aggregation in presence of epinephrine 4 μM, ionophore 5 μM, ADP 2,5 μM, arachidonic acid 1 mM, collagen 1 μg/ml, TRAP 10 μM; (B) Glycoprotein expression at baseline and (C) after platelet activation; (D) Fibrinogen binding after ADP activation; (E) Dense bodies content analysis; (F) ATP secretion evaluation; (G) Basal PS exposure (H) and after platelet activation. The PS induction ratio is calculated as the ratio of PS after stimulation to baseline. Statistical significance assessed by Mann-Whitney test. ∗ = *P* < .05.
