# Supplementary figures and images for: Platelet function studies in myeloproliferative neoplasms patients with Calreticulin or JAK2V617F mutation
Source: Res Pract Thromb Haemost. 2023 Jan 31;7(2):100060. doi: 10.1016/j.rpth.2023.100060 (PMC9992751; doi:10.1016/j.rpth.2023.100060)

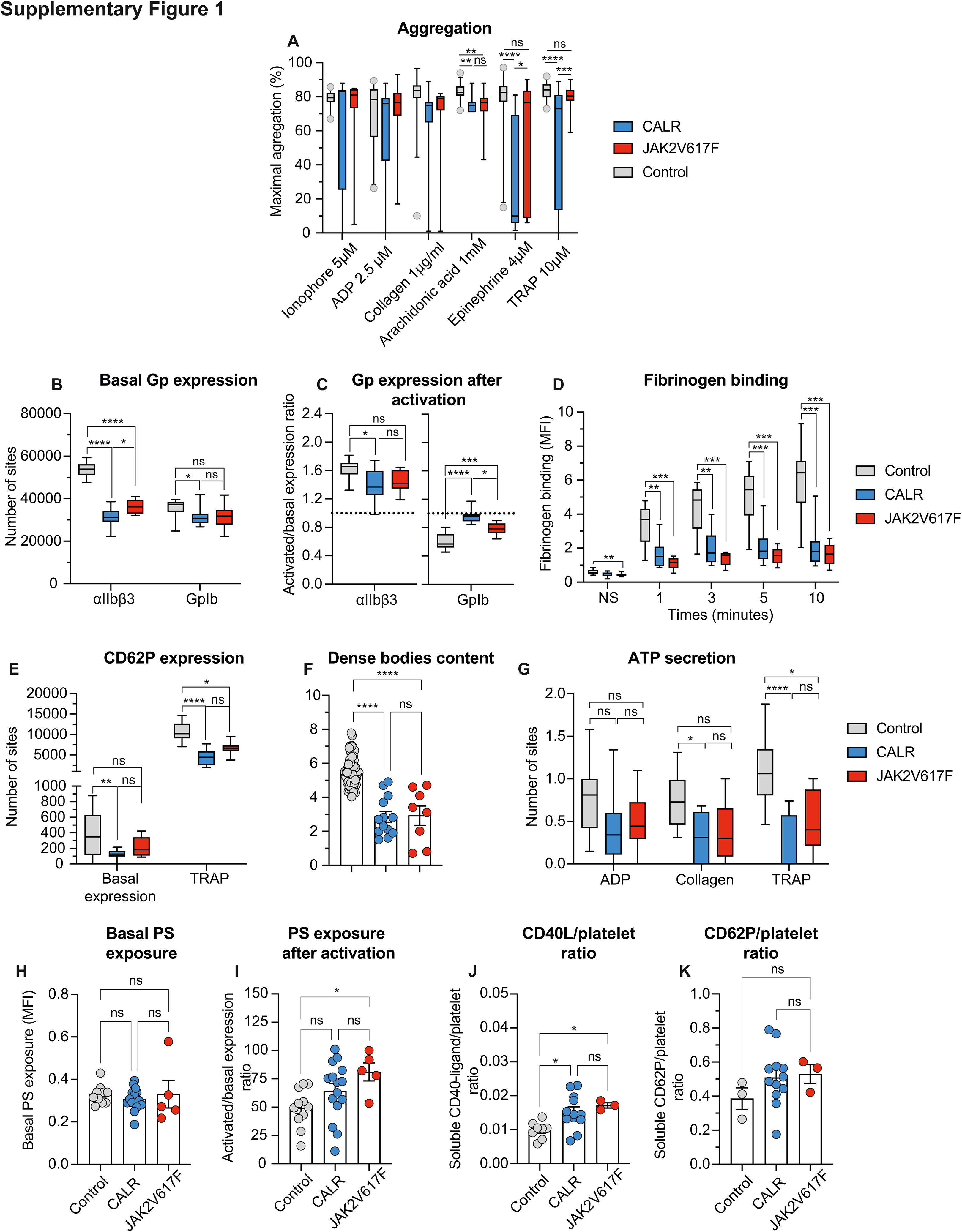

Supplement: Supplementary Figure 1 — Analysis of platelet function in ET patients. (A) Platelet aggregation in presence of epinephrine 4 μM, ionophore 5 μM, ADP 2,5 μM, arachidonic acid 1 mM, collagen 1 μg/ml, TRAP 10 μM; B) Glycoprotein expression at baseline and (C) after platelet activation; (D) Fibrinogen binding after ADP activation; (E) CD62P expression at basal level and after platelet activation; (F) Dense bodies content analysis; (G) ATP secretion evaluation; (H) Basal PS exposure (I) and after platelet activation. (J) CD40-L/platelet ratio analysis. Statistical significance assessed by Kruskal-Wallis test followed by post-hoc Dunn’s test. Data are presented as mean and SEM. (K) CD62-P/platelet ratio analysis. Statistical significance assessed by Kruskal-Wallis test followed by post-hoc Dunn’s test. Data are presented as mean and SEM. [file figs1.jpg]

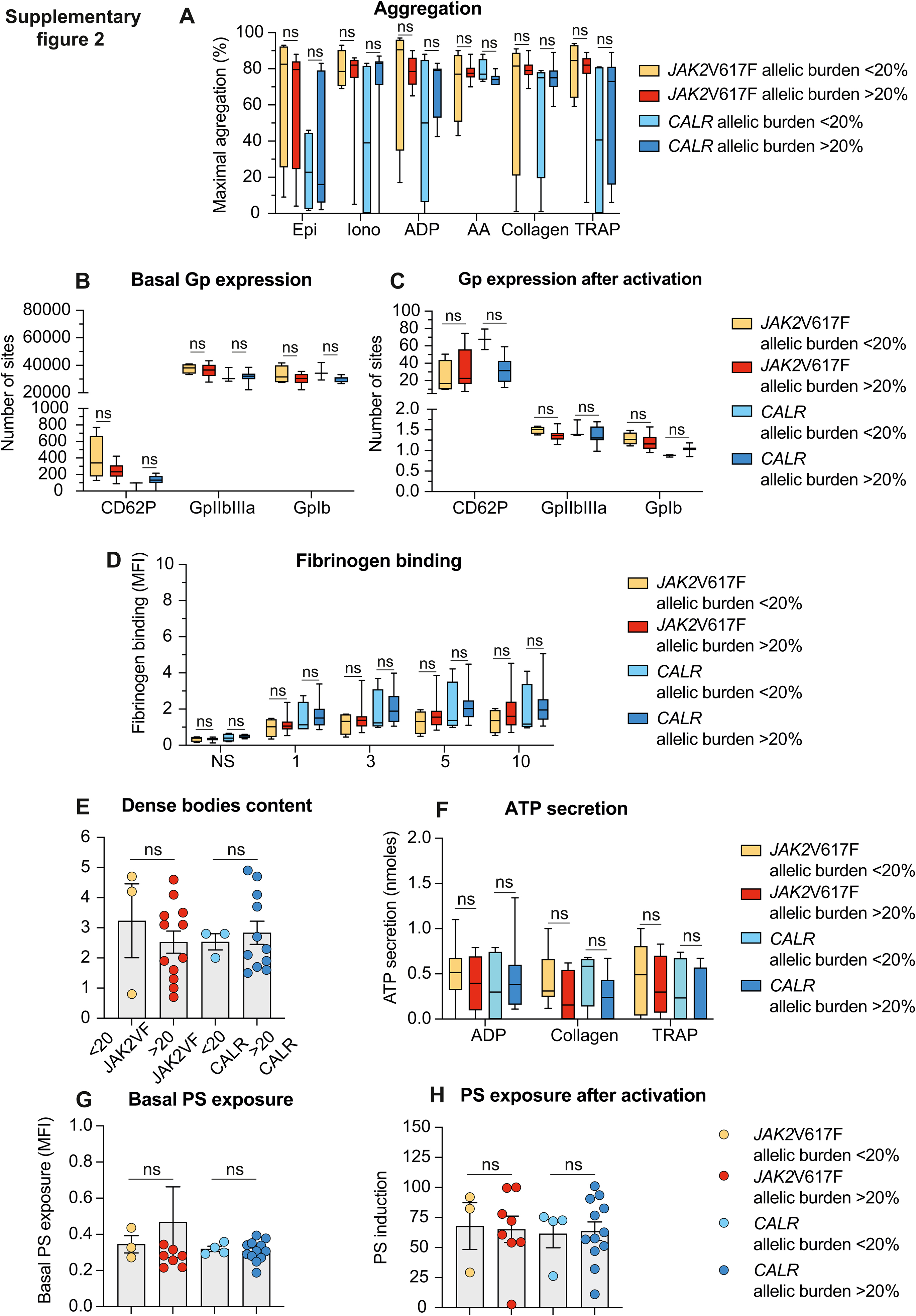

Supplement: Supplementary Figure 2 — Platelet function testing in patients with low or high allelic burden. (A) Platelet aggregation in presence of epinephrine 4 μM, ionophore 5 μM, ADP 2,5 μM, arachidonic acid 1 mM, collagen 1 μg/ml, TRAP 10 μM; (B) Glycoprotein expression at baseline and (C) after platelet activation; (D) Fibrinogen binding after ADP activation; (E) Dense bodies content analysis; (F) ATP secretion evaluation; (G) Basal PS exposure (H) and after platelet activation. The PS induction ratio is calculated as the ratio of PS after stimulation to baseline. Statistical significance assessed by Mann-Whitney test. ∗ = P < .05. [file figs2.jpg]
